# Supplementary figures and images for: Comparative Analysis of miRNA Expression Profiles under Salt Stress in Wheat
Source: Genes (Basel). 2023 Aug 4;14(8):1586. doi: 10.3390/genes14081586 (PMC10454085; doi:10.3390/genes14081586)

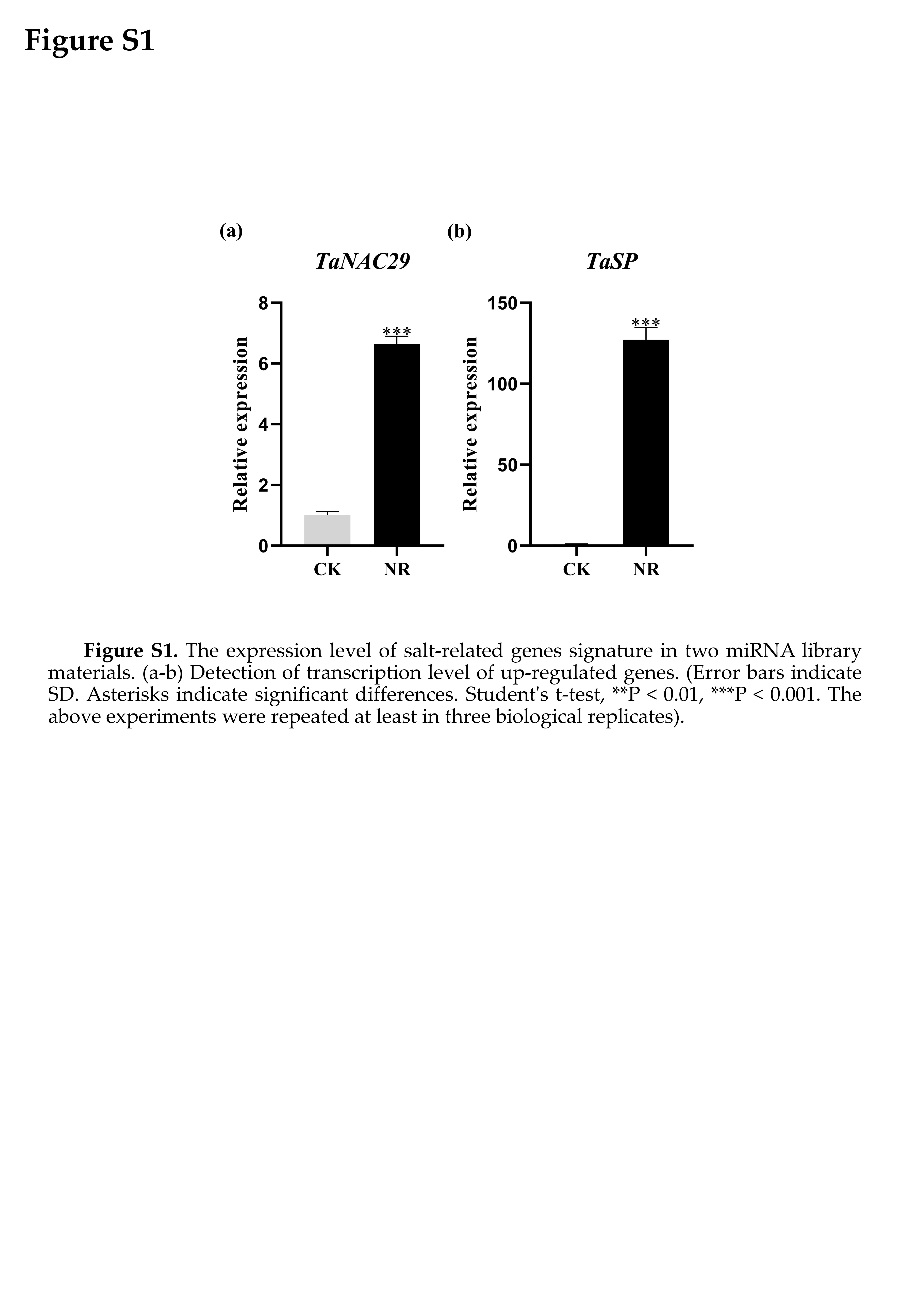

Supplement: Supplementary file 1 [file genes-14-01586-s001.zip › Figure S1.JPG]
